# Participation in environmental health research by placenta donation – a perception study

**DOI:** 10.1186/1476-069X-6-36

**Published:** 2007-11-22

**Authors:** Uffe Lind, Tina Mose, Lisbeth E Knudsen

**Affiliations:** 1 Institute of Public Health, University of Copenhagen. Øster Farimagsgade 5, P.O.B. 2099, 1014 Copenhagen K, Denmark

## Abstract

**Background:**

Much environmental health research depends on human volunteers participating with biological samples. The perception study explores why and how people participate in a placenta perfusion study in Copenhagen. The participation implies donation of the placenta after birth and some background information but no follow up.

**Methods:**

Nineteen semi-structured qualitative interviews were conducted with participants in the placenta perfusion study after donation of placenta. Observation studies were made of recruitment sessions.

**Results:**

The interviewed participants are generally in favour of medical research. They participated in the placenta perfusion study due to a belief that societal progress follows medical research. They also felt that participating was a way of giving something back to the Danish health care system. The participants have trust in medical science and scientists, but trust is something which needs to be created through "trust-work". Face-to-face interaction, written information material and informed consent forms play important parts in creating trusting relationships in medical research.

**Conclusion:**

Medical research ethics do not only amount to specific types of written information material but should also be seen as a number of trust making performances involving researchers as well as research participants.

## Background

The background of the perception study is a placenta perfusion study undertaken at the Institute of Public Health, University of Copenhagen. The placenta perfusion study uses placentas from women who have given birth by Caesarean section in studies of placental transport of substances from mother to foetus. Sections of the fresh placenta are immediately used in an experimental setup keeping the tissue functional and providing data regarding transport and background concentrations of study compounds of e.g. phthalates [1-3]. The women donating their placenta to the placenta perfusion study thereby participate in environmental health research. The participants in the placenta perfusion study who were interviewed for the perception study were asked to donate their placenta, when attending an information meeting at the hospital the day before giving birth. As part of the scheduled items in the information programme one of the researchers from the placenta perfusion study would enter the room and ask if the coming mothers would participate in the study while handing out written information material and informed consent forms. A poster informing about the project and asking all pregnant women to consider donation for the project is displayed in the clinic for pregnancy check up visited by all potential donors. This poster refers to the web page of the project providing in depth information about the study [4]. In all normal births the placenta is discarded as biological waste and regarded as biological tissue belonging to the mother. The coming mothers were asked to decide whether to participate or not and to read the written information material and fill out the consent form at the information meeting. The families were offered the opportunity to consider donation until the next morning where the Caesarean was scheduled. Most of the coming mothers participated in the study which may seem surprising considering the circumstances.

The overall research question of the perception study is: Why do people participate in the placenta perfusion study in particular and in environmental health research in general, and what are their perceptions of the ethical and communication practices in the placenta perfusion study? The aim of the perception study is twofold. 1. The study explores the donors' participation in the placenta perfusion study. Is this participation of any importance to the participants and if so, why and how? The perception study thereby also explores the perceived relevance of environmental health research in general and the placenta perfusion study in particular. On a more general level the perception study discusses how and why people participate in the production of medical knowledge and technologies. 2. The perception study also assesses whether the ethical and communication practices in the placenta perfusion project match the needs or expectations of the participants. For instance, do the participants think that they are informed too much or too little, how are the information procedures perceived, and what do participants make of informed consent forms?

There is an extensive sociological, anthropological and public health literature on the participation in medical research. Where much of this literature focuses on ethnicity [5-7] and on the participation of patients in research regarding the disease from which they suffer [8-10], this study focuses on the participation of healthy people in medical research, and ethnic origin is not ascribed any significance. Moreover, the published field research studies on the participation in environmental health research are few [11] and the field of research new [12,13].

The perception study is structured around the theoretical themes: motives, expectations, altered understandings of self, health and disease in relation to participation, and involvement in science.

These themes are inspired by science and technology studies (STS), which is a branch of the social sciences that studies the interdependencies of science and society. A central thesis in STS is that science and society are co-constructed [14,15]. This means that science is not seen as being outside society in any physical, ideological or epistemological sense. Science, according to the thesis of co-construction, is created in and by society and its members, which makes it possible to study science sociologically. In the mutual shaping of science and society, expectations – in this case the participants' expectations – are believed to play an important role [16]. However, the thesis on co-construction also stresses that science and research ultimately change society and people on many levels and in multiple ways. One of the ways might be that people are affected when participating in research, which in fact is the purpose of many intervention studies. An effect of the participation might be that participants see and understand certain phenomena in novel ways. Finally, by focusing on the social processes and the actors in scientific research, the thesis on co-construction makes it reasonable to study *how *people are involved and engaged in environmental health research and to study in what ways the ethical and communication guidelines are practiced and perceived.

## Methods

### Study design

The perception study used a method triangulation of observation studies and qualitative interviews. Observation studies were used to get insight into the practices of recruiting donors to the placenta perfusion study (see section on setting and data collection). Qualitative interviews were used to both qualify and problematise the theoretical perspectives of the perception study. When studying science and research from the perspective of STS, science and research become matters of social relations that need to be understood as much as explained, and thus require some kind of access to the perspectives of the actors involved, in this case the placenta donors. In comparison to other methods in the social sciences, observational studies and qualitative interviews are seen to be some of the best ways of getting access to the subject of the study through the privileged position and experiences of the actors involved [17]. Qualitative interviews may bring about personal and exclusive stories that can give a better understanding of the complexity of the subject matter, in this case the co-construction of the placenta perfusion study and the research participants. By being open to unexpected perspectives the qualitative interview makes it possible to become surprised by the informants and their perspectives. Researchers, including the sociologist engaging in qualitative studies, may perceive ethical and communication practices and problems in certain ways, but the practices and problems of ethics and communications may be of another kind when seen from the perspective of the participants.

### Setting and data collection

The research questions were operationalised into an interview guide of 48 questions. The interview guide was used to conduct 19 interviews with donors of placentas to the placenta perfusion study; 5 interviews with both mother and father, 13 interviews with just the mother and 1 interview with just the father. The donors participate in environmental health research at what would seem a very critical moment, namely just before giving birth through Caesarean section. This makes the donors and their involvement in environmental health research an "extreme and critical case" [18] in the study of research participation in that it might illustrate some general tendencies through extreme practices. If the donors for instance feel obliged to participate in medical research despite their critical situation this might say something general about the conditions for participating in medical research in Denmark. Giving birth through Caesarean section is an extreme practice in the sense that for most of the participants this was something unwanted and it involves surgery. Many of the participants therefore seemed to be anxious the day before giving birth. Nevertheless, most of them participated in the placenta perfusion study.

### Recruitment and ethics

The interviewed donors (informants) were recruited at the same time as being recruited to the placenta perfusion study. The recruitment to the placenta perfusion study took place at an information meeting at the hospital the day before the women gave birth. During the information meeting a researcher from the placenta perfusion study would address the possible donors and tell them about the study and ask whether they would want to participate, while handing out information material and informed consent forms. Following this, the possible donors would be asked to participate in the perception study by the sociologist. If so, they were asked to write down their name and number and they would then be contacted about a week after giving birth, and asked if they were still interested in participating in the perception study. Recruiting the informants for the perception study at the same time and place as the recruitment of donors to the perfusion study made it possible to do observation studies, but it may also have been a source of selection bias since the placenta perfusion study and the perception study could be seen as two parts of the same study, which was not the case. The informants signed informed consent forms stating that they would be anonymous to anyone except the interviewer. Permission to conduct the interviews, transcribe and store them was granted by the Danish Data Protection Agency (journal number 2007-41-0592). The participation does not imply any follow-up approaches from the research team.

As part of the recruitment strategy, the sociologist offered to interview the informants in their homes, and as a consequence all of the interviews were conducted in the homes of the informants. Interviews were conducted in Danish and range from 20–40 minutes in duration. Quotations in this article are translated into English by the authors. The interview guide included questions about motives, decision making process, practices of information, recruitment procedure, expectations, and relations between environment and health. The study did not include any systematic information about the participants' socio-economic status or educational level since it was not the aim of the perception study to do a representative study of the population. There are no interviews with people who did not wish to participate in the placenta perfusion study. According to the decisions of the Regional Ethics Committee a no must be respected and no other further approach is allowed. Characteristically the people saying no to donate their placenta also seemed to find it annoying or inappropriate to be addressed about other matters than that of their forthcoming birth.

Data collection was concluded after 19 interviews with participants in the perfusion study. The original rate of success was 15 interviews. Based on literature on qualitative methods as well as on personal experiences from former qualitative studies 15 in-depth interviews combined with observation studies were judged to give a comprehensive understanding of the social complexities of participating in the placenta perfusion study [19,20]. After conducting 19 interviews no new significant perspectives of the subject matter seemed to emerge. When transcribed into written material, the interviews amount to 230 pages.

### Analysis

The perception study uses an interactionist method of analysis. According to the interactionist perspective, the qualitative interview is seen as a certain social scientific practice with the aim of producing certain forms of knowledge [19]. The interactionist approach thus turns the STS thesis of co-construction on the knowledge production within the social sciences itself. The interview is thereby understood as a meeting between the researchers' theoretical perspectives and research questions and the personal stories and experiences of the informant, and the art of interviewing is to pursue certain research questions while at the same time being open to new and unanticipated perspectives [20]. In the study in question this means that the sociologist had an idea of what the interviews should be about, hence the interview guide about expectations, motivations, decision making process etc. The interviewer set the stage and guided the interview in the wished for directions. However, an important part of qualitative analysis in general and interactionist analysis in particular is being attentive to unforeseen issues during and after the interview. In this study this led to several unforeseen talks, and further on analysis, about for instance the relations between medical research and societal progression and the notion of giving something back to the Danish health care system.

The result of the interview is thus not the untouched perspective or hermeneutical truth of the informants' life-world but a negotiation of meaning and reality emerging through the practice of interviewing. When adopting the interactionist approach it would therefore be false to say that the methods of the perception study are either deductive *or *inductive. It seems more appropriate to characterise the practice of conducting and analysing qualitative interviews and observation studies as a continuous oscillation between deductive and inductive modes of knowing and reasoning. In practice this means that the research questions, the interview guide and the way in which the interviews with the donors from the perfusion study were conducted led to certain forms of knowledge situated in a particular space and time. On the one hand, it is important to take these contextual considerations into account when assessing the results of the perception study. On the other hand this does not mean that the perception study creates types of knowledge that can not lead to general conclusions.

## Results and discussion

The results of the analysis from the perception study is organised and presented in three parts: reasons for participating, trust in science, and ways of informing.

### Reasons for participating

This part of the analysis is based on the questions and answers about expectations and motivations in relation to the informants' participation in the placenta perfusion study. The overall perception of medical science and research expressed in the interviews is that it is important. When asked to elaborate on this, informants talked about how science and research is synonymous with societal progress and that it is a duty to participate in this progression. Two quotes by two informants illustrate these notions: "*I believe that it is very important to participate in studies of this kind. It advances research and the development, so I think it is a positive thing [...] It is a way in which an enlightened society can progress*" and: "*You can't just sit and be afraid in your little room. You have to collaborate and by doing that give something to the common good*". This sense of duty to participate must be understood in a Danish context. Since the Danish health care system is funded through taxes collected by the Danish state, health care in Denmark is often perceived as both free and as a collective project. Some of the informants thus stressed that participating was a way of giving something back to the Danish health care system. The participants' motivations to participate should also be analysed in relation to the expectations expressed in the interviews. These expectations were about how medical research changes society for the better and that this depends on the participation of "normal people". We may therefore conclude that making society a better place through environmental health research is seen by the informants as a co-operation between scientists and citizens.

The above mentioned motivations could be interpreted as altruistic in a universal sense of the concept, but then motivations are always located in a specific space and time and altruism should therefore be understood as an effect of local circumstances, rather than being a universal explanation. The importance of participating in the placenta perfusion study expressed in the interviews may thus be seen as a result of the direct relevance of this type of research to the donors. The participants easily related to the project since they were pregnant themselves and none of the informants saw themselves as persons seeking out participation in medical research as such.

Finally, an important reason for participating was that it was easy. Being asked the day before giving birth was, of course, troublesome for some of the interviewed participants. However, they all stressed that all it really took was to say yes and the placenta would change from being biological waste disposal to becoming raw material in environmental health research. The altruism of the participants should therefore also be seen as an effect of the easiness of participating.

### Trust in science

The participants' perception of the participation as a co-operation underlines the feelings of trust in the placenta perfusion study expressed in the interviews. The trust in the placenta perfusion study is twofold. 1. The informants expressed trust in the meaningfulness of the study. Without knowing many details about the study most of them decided that they would participate and donate their placenta. They also trusted that the researchers would do as they said they would and that the researchers would handle the placenta properly. 2. The informants also trusted that the ethical guidelines would be followed and that these guidelines would protect them sufficiently. Interviews and observation studies showed that far from all of the participants read the information material before signing the consent form, but that they trusted the researcher standing in front of them. One of the informants explains: *"You don't want to sit and read...you know, all that ethical stuff...you know what it says already, and you don't bother reading it through [...] And I think that it has something to do with that I trust that they are treating the placenta properly"*. The relation of trust may therefore be understood as an effect of the face-to-face interaction between the researcher and the participants and not something that solely emerges from reading the written information material and consent form.

This does not imply, however, that the informants found it superfluous to be asked and give consent. On the contrary, all informants, except one, stressed the importance of giving consent in some form. An informant makes this explicit: *"I think that it is fundamentally right that you give people the opportunity to say no, because it is in a sense intimate to some people [...] I think just being asked is important because one is already squeezed into a very efficient system such as the health care system. You feel like a piece being moved around, so it is nice that you sometimes feel that you have some rights and can say yes and no"*. The participants may not have read the information material or consent thoroughly but they nonetheless expressed a need for these documents to be there as a sign of the study being conducted properly. Borrowing from the language of theatre science we might say that written information material and consent forms are important props in the performance of trusting relations between the researchers and the participants. During the observation studies we also witnessed recruitment sessions with critical and clarifying questions posed by the possible participants before deciding to donate. This indicates that the participants did not trust the researchers passively but that trust needed to be produced collectively through "trust-work" where the face-to-face interaction between researcher and participants was crucial.

### Ways of informing

The analysis of the interviews and the observation studies has so far shown that the participants are in general favour of medical science and research, and that they trust scientists. However, possible participants still have to be made interested in the specific research project in question. In this process of making possible research participants interested the ways of informing seemingly play an important role. The fact that some of the participants did not read the information material and informed consent forms does not imply that they found information about the study unnecessary, but it implies that what was experienced as suitable ways of informing depended on the space and time in which information was given. According to the interviewed participants both written and oral information is important but the two ways of informing are perceived differently according to the circumstances. If possible, the written information material should be provided to the participants in advance. This would heighten its chances of being read. At the information meeting the oral information given by the researcher in the face-to-face interaction with the possible participants was in the forefront, whereas the written information and consent forms were perceived as mere tokens of the study being conducted properly.

When talking about the form and substance of both written and oral information the informants stressed that both types should be short and precise and that the purpose of the study should be at the very beginning. The informants wanted to know why they should participate in this particular study and some wanted to become interested and involved in the project. Several of the informants suggested that a way of involving participants and make them interested would be by informing them about already obtained results from the study. One of the informants explains: *"You can "sell" the project even better if you tell about already obtained results. You could have said something about some known and debated substance that you had found goes from the mother to the child"*. The informants were asked if they would the visit the placenta perfusions study website in the future to follow the study and know more about the results. About half of the informants said that they might do that.

When we understand recruitment as a matter of involvement rather than persuasion it underlines the importance of giving participants an opportunity to ask questions and making suggestions. All of the informants explained that this was essential in their decision to donate, here exemplified by one of the mothers: *"If you are not sure, and there are some things that you want to get clear, then it is important that you have the opportunity to ask some questions before you decide. I think that should always be the case"*. Informants explained that a project would seem less trustworthy if there was no time or space for asking questions. The mere opportunity for asking questions and making suggestions thus signals openness and creates an atmosphere of trust. The involvement and engagement of the participants also shows that they wished to learn from the participation. Some had specific questions about methods or already obtained results which they posed at the information meeting or during the perception study interview, while others expressed a wish to know the results when the perfusion study ends. When asked whether it was negative or positive that participation might have an effect on the ways in which participants think about environment and health in relation to their own lives all of the informants answered that this was positive. They wanted to learn and assess their own ways of living in the light of the research in which they participated.

Medical ethics has traditionally focused on the protection of the autonomy and dignity of research participants. Medical ethics may therefore have lacked focus on how to involve and engage research participants properly and in novel ways in the research projects in which they participate. The interviewed research participants in the placenta perfusion study are in general pro-science and they wished to participate, know results, ask questions and discuss methods. Some of the participants, it must be added, did not care about these matters and participated passively. This calls for flexible and pragmatic research, ethical and information practices that are able to take into account the different ways of participating in research. Table 1 summarises the results of the analysis.

**Table 1:** 

Reasons for participating	Trust in science	Ways of informing
The importance of medical research	Trust in the meaningfulness of the placenta perfusion study	Timing of information
Belief in societal progress following science	Trust in researchers and their compliance with ethical standards	Time and place for questions
Giving something back to the health care system	The importance of face-to-face-interaction	Short and precise information
The easiness of participating	The relation between information and trust	Information about previous results

## Conclusion

The perception study shows that people participated in the placenta perfusion study because they believed that participating in medical research in general and in the placenta perfusion study in particular would contribute to societal progress through improvements of the health of the population. Improving the health of the population is thus seen as a common project. The participants also felt that participating was a way of giving something back to the Danish health care system, and finally they participated because it was easy. Participants trusted the researchers from the placenta perfusion project and they expressed general trust in medical science and research. The perception study analysis underlines that trust is a social relation that needs to be performed. Face-to-face interaction, written material and informed consent forms, and time for critical and clarifying questions are important properties in the performance of trusting relationships in medical research involving human participants, although the participants may not even read the written information material. According to the interviewed participants the written information material should be short and precise and in both the written and oral material the purpose of the study should be in the forefront. Despite general trust in medical research the participants wished to know why they should participate in this particular study. Oral and written information play different parts according to the circumstances in which they are given. The analysis shows that ethics is not only about the right amount and style of written information material but also about a number of performances and affective relations between humans. In doing trust-work researchers have to put themselves at stake and navigate in the waters of the specific concerns that their research may create.

## Competing interests

The author(s) declare that they have no competing interests.

## Authors' contributions

UL carried out study design, interviews, and analysis of interviews and co-drafted the manuscript. LEK carried out study design and co-drafted the manuscript. TN organised the recruitment and co-drafted the manuscript. All authors have read and approved the final manuscript.
